# The *Trypanosoma brucei* tRNA methylome

**DOI:** 10.1128/mra.00579-24

**Published:** 2024-12-10

**Authors:** Kevin T. Militello

**Affiliations:** 1 Biology Department, State University of New York at Geneseo, Geneseo, New York, USA; University of Maryland School of Medicine, Baltimore, Maryland, USA

**Keywords:** 5-methylcytosine, epitranscriptome, bisulfite sequencing, tRNA

## Abstract

Herein, the *Trypanosoma brucei* procyclic form and bloodstream form tRNA methylome is reported as revealed by small RNA bisulfite sequencing. 5-Methylcytosines were identified at six unique positions with 54 total 5-methylcytosines revealed. The main hot spot for 5-methylcytosine in tRNA is the junction between the variable region and the T-arm.

## ANNOUNCEMENT

*Trypanosoma brucei* causes African Sleeping Sickness in humans and nagana in cattle (1). Our group performed sodium bisulfite sequencing (BS-seq) on *T. brucei* rRNA to map the positions of 5-methylcytosines (2) and reported the presence of two 5-methylcytosines in the large rRNA (3). There is little known about 5-methylcytosine in other *T. brucei* RNAs (4).

*T. brucei* 29–13 procyclic form (insect) parasites were grown at 27°C in SM media with 10% fetal bovine serum (FBS) (5). Bloodstream form 427 (single-marker) parasites were grown at 37°C in HMI-9 media containing 10% FBS (6). Insect/animal hosts were not necessary. These parasite lines were genetically modified for RNAi experiments > 20 years ago and have been maintained via *in vitro* culture (7). These strains are considered “wild-type” parasites for experiments. *Escherichia coli* wild-type strain BW25113 was grown in LB at 37°C with shaking at 250 RPM until the stationary phase (OD_600_ ~ 3.0). *T. brucei* total RNA was isolated using TRIzol (Thermo Fisher Scientific); *E. coli* total RNA was isolated using the MasterPure kit (Epicentre). Total RNAs were treated with DNA-free DNase (Invitrogen). Small RNA fractions were enriched from total RNA using the RNA Clean and Concentrator kit (Zymo Research). Small RNA samples were treated with sodium bisulfite for four cycles using the Epitect Bisulfite kit (Qiagen) (3). TruSeq small RNA libraries were generated and sequenced on an Illumina HiSeq 2500 machine (50 cycles) at the University at Buffalo Genomics and Bioinformatics Core (Buffalo, NY). Bioinformatic analyses were performed in Galaxy (8); default parameters were used for all manipulations. Illumina adapters, sequences < 20 basepairs, and sequences < 20 PHRED scores were removed with Trim Galore v0.67 (9). Reads were mapped to species-specific “tRNA genomes” containing one copy of each tRNA isoacceptor using Bismark/Bowtie v0.22.1 (10) (files available at figshare.com). 5-Methylcytosine positions were called with MethylExtractor v0.22.1 (10). Cytosines with <10× coverage and cytosines on the minus strand were removed. Two different RNA preparations per sample were sequenced (*n* = 2); see Table 1.

**TABLE 1:** Small RNA bisulfite sequencing samples

Organism	*E. coli*	*E. coli*	*T. brucei*	*T. brucei*	*T. brucei*	*T. brucei*
Stage	Stationary	Stationary	Procyclic	Procyclic	Bloodstream	Bloodstream
Replicate	1	2	1	2	1	2
SRA accession number	SRX24722605	SRX24722606	SRX24722453	SRX24722454	SRX24722455	SRX24722456
Reads	19965735	22570943	23048971	17633438	17456157	21105417
^*a*^Mapping efficiency	60.4%	59.4%	36.4%	25.9%	26.5%	25.6%
Cytosines queried	921	926	904	888	891	891
Median fold coverage	3,787	3,909	460	431	458	534

^*a*^ To tRNA genes only (tRNA genome).

**Fig 1:**
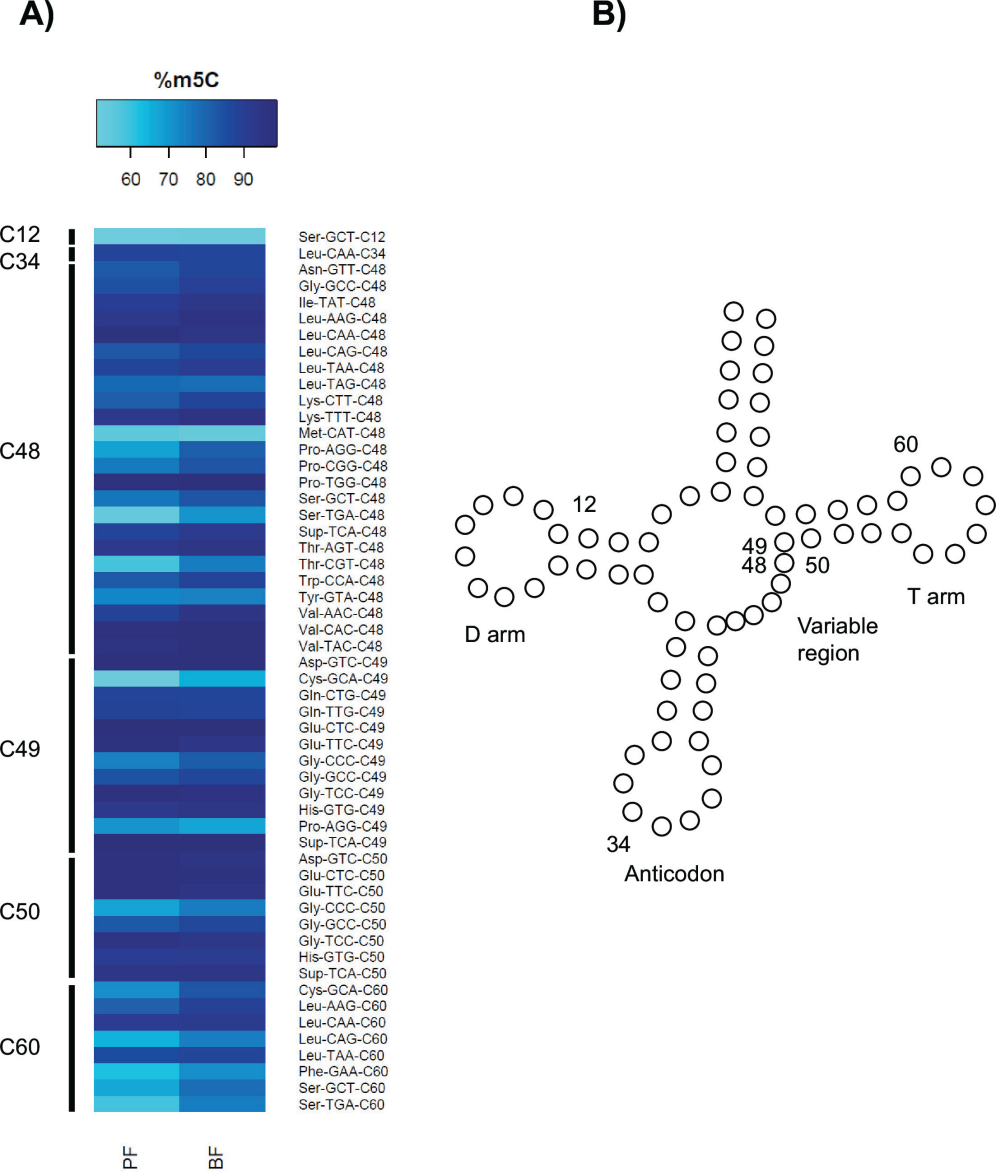
(**A**) Heat map of cytosines with >50% methylation in *T. brucei* tRNA sorted by tRNA position. PF is procyclic form tRNA and BF is bloodstream form tRNA. (**B**) Basic tRNA structure based on tRNA^Lys(CTT)^ with reported cytosines labeled.

First, *E. coli* tRNAs were analyzed as a control; they lack 5-methylcytosine (11, 12). The *E. coli* tRNA deamination rates averaged 90.6%, indicating good conversion of non-5-methylcytosines to uracils necessary for identifying the deamination-resistant 5-methylcytosines. Next, *T. brucei* tRNAs were analyzed (Fig. 1). Cytosines with >50% 5-methylcytosine in both samples were called “5-methylcytosines” according to a stringent cut-off (3, 11). Fifty-four 5-methylcytosines were identified in *T. brucei* tRNA at six positions (0–3 5-methylcytosines per tRNA). 5-Methylcytosines were present in procyclic and bloodstream form tRNA; there were no statistically significant differences (two sample *t*-tests, *P* > 0.05). The most common 5-methylcytosine positions were at the variable region/T-arm junction (C48-C50). This position has been reported in other eukaryotes (11–16) and in our small report for selected *T. brucei* tRNAs (4). Evidence for 5-methylcytosine at C34 in the tRNA^Leu(CAA)^ anticodon was detected and is found in other eukaryotes (11, 12, 15, 16). There is evidence for methylation of additional sites including C60 (12). The data sets provide a framework for elucidating *T. brucei* 5-methylcytosine function in tRNAs and other small RNAs.

## Data Availability

The data are present and available in NCBI BioProject PRJNA996772. See Table 1 for specific Sequence Read Archive accession numbers.
